# Green space, air pollution, traffic noise and saliva cortisol in children: The PIAMA study

**DOI:** 10.1097/EE9.0000000000000141

**Published:** 2021-04-02

**Authors:** Lizan D. Bloemsma, Alet H. Wijga, Jochem O. Klompmaker, Gerard Hoek, Nicole A. H. Janssen, Marieke Oldenwening, Gerard H. Koppelman, Erik Lebret, Bert Brunekreef, Ulrike Gehring

**Affiliations:** a National Institute for Public Health and the Environment (RIVM), Bilthoven, The Netherlands; b Institute for Risk Assessment Sciences (IRAS), Utrecht University, Utrecht, The Netherlands; c Department of Pediatric Pulmonology and Pediatric Allergology, Beatrix Children’s Hospital, UMCG, GRIAC Research Institute, University of Groningen, Groningen, The Netherlands; d Julius Center for Health Sciences and Primary Care, University Medical Center Utrecht, Utrecht, The Netherlands.

## Abstract

Green space, air pollution, and traffic noise exposure may be associated with stress levels in children. A flattened diurnal cortisol slope (the decline in cortisol concentrations from awakening to evening) is an indicator of chronic stress. We examined associations of green space, ambient air pollution, and traffic noise with the diurnal cortisol slope in children 12 years of age.

**Methods:**

At age 12 years, 1,027 participants of the Dutch PIAMA birth cohort collected three saliva samples during 1 day. We estimated residential exposure to green space (i.e., the average Normalized Difference Vegetation Index [NDVI] and percentages of green space in circular buffers of 300 m and 3,000 m), air pollution, and traffic noise. Associations of these exposures with the diurnal cortisol slope (in nmol/L per hour) were assessed by multiple linear regression, adjusting for potential confounders.

**Results:**

Higher average NDVI and total percentage of green space in a 3,000 m buffer were associated with a larger diurnal decrease in cortisol levels (adjusted difference [95% confidence interval] = −0.11 nmol/L/hr [−0.21, 0.00 nmol/L/hr] per interquartile range increase in the average NDVI; −0.13 nmol/L/hr [−0.26, 0.00 nmol/L/hr] per interquartile range increase in the total percentage of green space). These associations were largely driven by associations with the percentage of agricultural green space and by associations in children living in nonurban areas. We observed no relationships between air pollution or traffic noise and the diurnal cortisol slope.

**Conclusions:**

Residential exposure to green space in a buffer of 3,000 m may be associated with lower stress levels in children 12 years of age.

## Abstract 2

Supplemental Digital Content is available in the text.

**What this study adds**

In daily life, people are exposed to multiple environmental risks (such as air pollution and noise) and environmental amenities with potentially positive health effects (such as green space). We examined the individual and joint associations of green space, ambient air pollution, and traffic noise with the diurnal cortisol slope, an indicator of chronic stress, in children 12 years of age in the Netherlands. This study shows that residential exposure to green space in a buffer of 3,000 m may be associated with lower stress levels in children 12 years of age. We found no relationships between air pollution or traffic noise and the diurnal cortisol slope.

## Introduction

Green space may improve health by reducing stress, promoting physical activity, increasing social cohesion, and reduced exposure to environmental stressors, including air pollution and noise.^1–3^ Previous studies have shown associations between exposure to green space and reduced stress levels, mainly in adults.^4–7^ In contrast, exposure to ambient air pollution and traffic noise may be associated with higher stress levels.^8–12^

The secretion of the stress hormone cortisol follows a circadian rhythm, characterized by high levels upon awakening, a substantial increase in cortisol concentrations in the 30–45 minutes after awakening (the cortisol awakening response [CAR]), followed by declining cortisol concentrations until reaching its minimum around bedtime.^13,14^ The diurnal slope is the change in cortisol concentration from the post-awakening peak to its lowest point. Stress activates the hypothalamic–pituitary–adrenal (HPA) axis and the subsequent release of cortisol. With repeated stress exposure, the HPA axis becomes less flexible, which results in smaller differences between morning and evening cortisol concentrations.^15^ Several studies have shown relationships between long-term stress exposure and a flattened diurnal cortisol slope.^13,16^ Additionally, it has been suggested that the diurnal cortisol slope is a superior predictor of both chronic stress and potential HPA axis dysregulation compared to other measures of cortisol, such as the total daily cortisol output.^13^

The associations of green space, air pollution, and traffic noise with cortisol levels in children and adolescents are not clear as the few epidemiologic studies so far have reported inconsistent results. Van Aart et al^17^ found no relationship between residential greenness and hair cortisol concentrations, an indicator of stress exposure during the last 3 months, in 153 children in Belgium. Few studies found higher urinary or saliva cortisol concentrations in children exposed to high levels of road traffic noise.^9^ A recent study, however, found no associations between residential exposure to road traffic noise and saliva cortisol concentrations in 1,751 adolescents in Stockholm County.^18^ One study found that higher exposure to nitrogen dioxide (NO_2_) was associated with a decrease in the diurnal cortisol slope in 140 adolescents in California.^19^ To the best of our knowledge, no previous epidemiologic studies have assessed relationships between green space or traffic noise and the diurnal cortisol slope in children or adolescents.

Exposures to green space, air pollution, and traffic noise are generally spatially correlated. Road traffic is a major source of both air pollution and noise, while higher levels of green space are associated with lower levels of air pollution and noise.^20–23^ It is therefore important to assess both the individual and joint associations of these exposures with health outcomes in children. The aim of this study was to examine the individual and joint associations of green space, ambient air pollution, and traffic noise with the diurnal cortisol slope, an indicator of chronic stress, in children 12 years of age.

## Methods

### Study design and population

This study was performed within the ongoing Dutch Prevention and Incidence of Asthma and Mite Allergy (PIAMA) birth cohort study. Detailed descriptions of the PIAMA study have been published previously.^24,25^ Briefly, pregnant women were recruited in 1996/1997 from the general population in three different parts of the Netherlands: regions north, central, and west (Figure S1; http://links.lww.com/EE/A123). Region central includes the provinces of Utrecht, Gelderland, and Flevoland, and region west contains the city of Rotterdam and surrounding municipalities. Region north (provinces Friesland, Groningen, and Drenthe) has a lower population density, higher green space levels, and substantially lower air pollution and traffic noise levels than regions central and west. The baseline study population consisted of 3,963 children. Participating parents completed questionnaires during pregnancy, 3 months after their child was born, when the child was 1 year old, and yearly thereafter until the child was eight. When the children were 11, 14, and 17 years old, both parents and children completed questionnaires.

Additionally, 1,094 children (27.6% of the baseline study population) collected saliva samples as part of a medical examination at age 12 years, that took place from 2008 to 2010. For 24 children, at least one sample did not contain enough saliva to determine saliva cortisol concentrations. Furthermore, we excluded three participants from the present analysis because their samples were unintentionally mixed during analysis or their tubes did not have a cap. In addition, we excluded three children with diabetes, five children with extremely high cortisol concentrations (>3 SDs above the maximum of all other measured cortisol concentrations), and 32 children who used (inhaled) corticosteroids on the day of the saliva collection. Our study population consisted of 1,027 children (Figure S2; http://links.lww.com/EE/A123). The study protocol has been approved by the ethical review boards of the participating institutes and all parents and children gave written informed consent.

### Saliva cortisol

Children were asked to collect three saliva samples on one school day as part of a medical examination at age 12 years: immediately after awakening, 30 minutes after awakening (when cortisol levels are usually highest), and at 8.00 pm (in the evening). Children were not allowed to eat, drink or brush their teeth between the collection of the first and second samples. Participants were instructed to store the saliva samples in a refrigerator until a research assistant collected them, typically within a few days. The samples were then stored at −20 °C for 8–10 years until they were analyzed using the DEMEDITEC Cortisol free in Saliva ELISA kit from Demeditec Diagnostics GmbH (Kiel, Germany). Since we only had access to 1 day of saliva cortisol data, we assumed that the collection day represented a typical circadian cycle for the participants and that our observed associations reflect long-term effects.

Researchers have argued for excluding the CAR when calculating the cortisol slope, because the CAR is influenced by different biologic mechanisms than the rest of the diurnal cortisol rhythm.^14^ The cortisol concentrations immediately after awakening were therefore not included in the present analysis. Except for the CAR, the diurnal cortisol rhythm is characterized by a gradual decline throughout the day.^13^ We therefore calculated the diurnal cortisol slope in nmol/L per hour as follows: (cortisol concentration at 8.00 pm—cortisol concentration 30 minutes after awakening)/number of hours between the collection of the two saliva samples.

### Exposure assessment

We estimated exposure to green space, air pollution, and traffic noise at the children’s current home addresses at the time of the saliva collection at age 12 years. More details of the exposure assessment have been described elsewhere.^22,23^

#### Green space

The Normalized Difference Vegetation Index (NDVI) was used to assess greenness levels surrounding the children’s homes. The NDVI was derived from Landsat 5 Thematic Mapper data at a spatial resolution of 30 m × 30 m. NDVI values range from −1 to +1, with higher values indicating more greenness.^26^ A NDVI value of zero means no green vegetation and negative values correspond to water. We set negative NDVI values to zero so that the effects of water surfaces do not negate the presence of green space. We combined cloud-free images of the summer of 2010 to create a map of the Netherlands. We quantified residential surrounding greenness as the average NDVI in circular buffers of 100 m, 300 m, 500 m, 1,000 m, and 3,000 m around each participant’s home address. Since we observed high correlations between the average NDVI in different buffer sizes (Table S1; http://links.lww.com/EE/A123), we only included the average NDVI in buffers of 300 m and 3,000 m in the main analyses.

We additionally used “Bestand Bodemgebruik” of 2006, a detailed land-use map of the Netherlands, to assess the total percentage of green space and percentages of urban, agricultural, and natural green space in buffers of 300 m and 3,000 m around the children’s homes.^27^ Bestand Bodemgebruik divides the Netherlands into polygons with different classes of land-use, and is based upon the digital topographic map of the Kadaster with a scale of 1:10,000. Polygons that correspond to green space have a minimum size of 0.1 (for allotments) or 1 hectare. In contrast to the NDVI, Bestand Bodemgebruik does not include private green property (such as gardens) and street greenery. We assessed surrounding greenness and the percentages of green space in ArcGIS 10.2.2 (Esri, Redlands, California).

#### Air pollution

We estimated annual average concentrations of particulate matter with diameters of <10 μm (PM_10_) and <2.5 μm (PM_2.5_), PM_2.5_ absorbance, NO_2,_ and the oxidative potential of PM_2.5_ (electron spin resonance [OP^ESR^] and dithiothreitol [OP^DTT^]) at the children’s homes with land-use regression (LUR) models that were developed within the ESCAPE project. Models were based on air pollution monitoring campaigns that were performed between February 2009 and February 2010 in the Netherlands/Belgium. Details of the LUR model development have been published previously.^28–30^ The performance of the LUR models was evaluated using leave-one-out cross-validation (R^2^_LOOCV_) and ranged from 0.47 for OP^DTT^ to 0.89 for PM_2.5_ absorbance.^28–30^

#### Traffic noise

We used the Standard Model Instrumentation for Noise Assessments, developed at the Dutch National Institute for Public Health and the Environment, to estimate annual average road traffic and railway noise exposure.^31^ Daily average (L_den_) and nighttime average (L_night_) road traffic and railway noise exposure at the children’s home addresses were estimated for 2011. L_den_ is the A-weighted noise level over a whole day weighted with 5 dB(A) extra in the evening (19.00–23.00) and 10 dB(A) extra at night (23.00–7.00). We only included L_den_ in our analyses, because L_den_ and L_night_ were highly correlated (r = 0.99 for road traffic noise; r = 0.95 for railway noise).

### Potential confounding variables

We obtained information on parental level of education, maternal smoking during pregnancy (yes/no) and any smoking in the child’s home at age 11 years (yes/no) from parental questionnaires. We defined parental level of education as the maximum of the father’s and mother’s educational level, categorized as low/intermediate (primary school only, lower secondary or lower vocational education, intermediate vocational education, or intermediate or higher secondary education) and high (higher vocational education or university). A child had a high level of parental education if either his/her mother or father was highly educated. Children’s height (in centimeters) was measured by trained staff during the medical examinations at age 12 years. Pubertal development (using the puberty development scale: 1 = not yet started; 2 = barely started; 3 = definitely started; and 4 = seems complete) was reported by the children in a questionnaire administered at age 11 years.^32^ We used the status scores of the four-digit postal code areas from the Netherlands Institute for Social Research (SCP) of 2010 as an indicator of neighborhood socioeconomic status (SES). Four-digit postal code areas in the Netherlands range from approximately 1.1–8.3 km^2^ and comprise on average 2,106 households. Status scores comprise the average income, the percentage of residents with a low income, percentage of unemployed persons, and the percentage of low-educated residents in a postal code area. A lower status score indicates a lower neighborhood SES.^33^ Finally, we used the average number of addresses per km^2^ of the four-digit postal code areas to assess the degree of urbanization. The average number of addresses per km^2^ is the standard and commonly used indicator of urbanicity in the Netherlands and has been provided by Statistics Netherlands (Centraal Bureau voor de Statistiek; CBS): https://www.cbs.nl/en-gb/news/2007/07/young-people-live-in-cities-children-in-rural-areas/degree-of-urbanisation. CBS defines four-digit postal code areas with an average of ≥1,500 addresses/km^2^ as strongly urbanized or extremely urbanized. We therefore included the degree of urbanization in two categories in our analyses: urban area (≥1,500 addresses/km^2^) and nonurban area (<1,500 addresses/km^2^).

### Statistical methods

We assessed the shapes of the unadjusted associations of the continuous exposures and potential confounders with the diurnal cortisol slope by generalized additive models with integrated smoothness estimation and an identity link (GAM function; The R Project for Statistical Computing 2.8.0, www.r-project.org). The associations of green space, air pollution, and traffic noise with the diurnal cortisol slope were linear or almost linear (Figure S3; http://links.lww.com/EE/A123). We therefore included all exposures as continuous variables in our multiple linear regression analyses with the diurnal cortisol slope (in nmol/L/hr) as dependent variable and expressed the associations per interquartile range (IQR) increase in exposure. Since 82.7% of the children had no natural green space in a buffer of 300 m around their homes, we created a binary variable: natural green space in a buffer of 300 m yes/no. We mutually adjusted the associations with the percentages of agricultural, natural, and urban green space in all analyses. Finally, we explored confounding of associations with one exposure by the other exposures of interest with three-exposure models. To ensure that we did not violate the independence assumption, the presence of spatial autocorrelation was investigated. We observed no spatial autocorrelation in the distribution of the diurnal cortisol slope (Moran’s I = −0.008, *P* = 0.529).

We specified several regression models with increasing degree of adjustment for potential confounders. Model 1 was the unadjusted model. Model 2 was adjusted for age, sex, parental level of education, maternal smoking during pregnancy, any smoking in the child’s home, pubertal development, height, and season. We then explored potential confounding by neighborhood SES (model 3) and degree of urbanization (model 4), by additionally adjusting for these variables. We evaluated effect modification by parental level of education by including a product interaction term between level of education and all exposures in adjusted models (model 4). We included the degree of urbanization in our analyses to account for the fact that people living in urban areas may experience more stress (for reasons other than air pollution, traffic noise, or lack of green space) than people living in nonurban areas.^34^ This could, however, lead to over-adjustment because the degree of urbanization is also a determinant of the exposures of interest, that is, green space, air pollution, and traffic noise. We therefore included the degree of urbanization in a separate model. For the average NDVI and total percentage of green space, we additionally examined effect modification by degree of urbanization by including a product interaction term between the degree of urbanization (in two categories: urban and nonurban) and the exposures in adjusted models (model 4).

We performed several sensitivity analyses. We assessed associations of green space in buffers of 500 m and 1,000 m with the diurnal cortisol slope (only for model 4). Since there was not enough variation in green space exposure in buffers of 100 m (58.4% of the children had no green space in a buffer of 100 m), we did not assess associations with green space in buffers of 100 m. It is possible that associations of green space, air pollution, and traffic noise with the diurnal cortisol slope become apparent only after longer cumulative exposure. We therefore excluded children who had moved in the 2 years preceding the collection of the saliva samples (n = 89) and repeated the analyses in the subgroup of non-movers. Finally, we excluded children whose saliva samples had not been stored according to protocol (n = 106): (1) samples that were stored at room temperature for a few days, (2) samples that had not been stored in the freezer within 7 days, and (3) samples that were unintentionally removed from the freezer for 24 hours. We performed the statistical analyses, except the GAM analyses of the linearity of the associations, with SAS version 9.4 (SAS Institute Inc., Cary, North Carolina).

## Results

**Figure 1.**
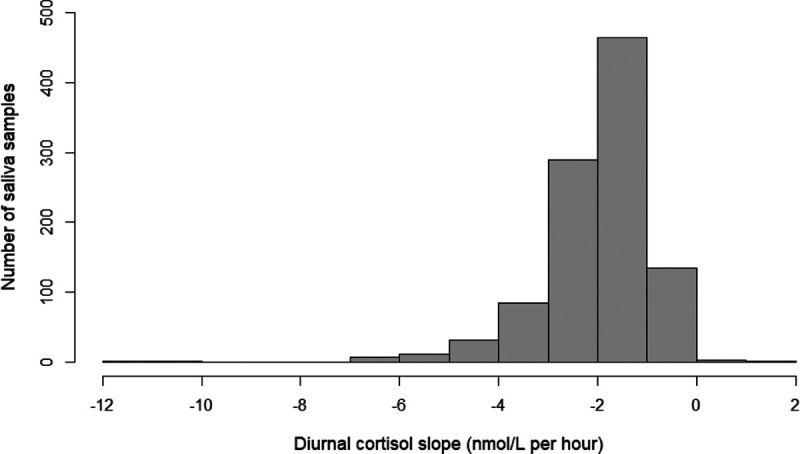
Distribution of the diurnal cortisol slope (in nmol/L per hour) in 1,027 children aged 12 years.

The median age of the study participants was 12.6 years (Table 1). A higher proportion of our study participants had highly educated parents and a lower proportion of mothers had smoked during pregnancy (13.3% vs. 17.9%) compared to the baseline PIAMA study population (n = 3963; Table S2; http://links.lww.com/EE/A123). The distributions of the diurnal cortisol slope and morning and evening saliva cortisol levels in our study population are shown in Figure 1 and Figure S4; http://links.lww.com/EE/A123. The median saliva cortisol concentration thirty minutes after awakening was 24.7 nmol/L (25th, 75th percentiles: 17.7, 33.4 nmol/L; Table 1). Evening cortisol concentrations were considerably lower with a median of 2.0 nmol/L (25th, 75th percentiles: 1.4, 2.8 nmol/L). The median diurnal cortisol slope was −1.8 nmol/L/hr (25th, 75th percentiles: −2.5, −1.3 nmol/L/hr).

**Table 1.** Characteristics of the study population and the distribution of green space, air pollution, and traffic noise levels.

Characteristic	n (%) or median (25th to 75th percentiles)
N	1,027
Saliva cortisol at waking (nmol/L)	16.4 (12.3 to 21.5)
Saliva cortisol 30 min post waking (nmol/L)	24.7 (17.7 to 33.4)
Saliva cortisol at 8.00 pm (nmol/L)	2.0 (1.4 to 2.8)
Diurnal cortisol slope (nmol/L/h)	−1.8 (−2.5 to −1.3)
Boys	504 (49.1)
Age (y)	12.6 (12.4 to 12.8)
Parental level of education	
Low/intermediate	426 (41.7)
High	596 (58.3)
Maternal smoking during pregnancy (yes)	135 (13.3)
Smoking in child’s home (yes)	111 (11.0)
Puberty development scale	1.4 (1.0 to 1.8)
Neighborhood SES^a^	0.29 (−0.38 to 1.09)
Degree of urbanization	
Urban area (≥1,500 addresses/km^2^)	418 (40.8)
Nonurban area (<1,500 addresses/km^2^)	607 (59.2)
Average NDVI in 300 m	0.55 (0.49 to 0.62)
Total percentage of green space in 300 m	12.4 (2.4 to 29.6)
Percentage urban green in 300 m	0.7 (0.0 to 7.4)
Percentage agricultural green in 300 m	0.2 (0.0 to 21.4)
Percentage natural green in 300 m	0.0 (0.0 to 0.0)
Buffers that have no natural green	846 (82.7)
Average NDVI in 3,000 m	0.63 (0.56 to 0.69)
Total percentage of green space in 3,000 m	56.3 (40.0 to 71.3)
Percentage urban green in 3,000 m	2.7 (0.9 to 4.7)
Percentage agricultural green in 3,000 m	44.1 (25.1 to 61.9)
Percentage natural green in 3,000 m	3.7 (1.5 to 10.4)
NO_2_ (µg/m^3^)	22.8 (18.1 to 26.6)
PM_2.5_ absorbance (10^−5^/m)	1.2 (1.0 to 1.3)
PM_10_ (µg/m^3^)	24.5 (24.0 to 25.0)
PM_2.5_ (µg/m^3^)	16.5 (15.6 to 16.7)
OP^ESR^ (A.U./m^3^)	932.8 (780.2 to 1026.4)
OP^DTT^ (nmol DTT/min/m^3^)	1.1 (1.0 to 1.2)
Road traffic noise (L_den_ dB(A))	52.2 (49.2 to 56.1)
Railway noise (L_den_ dB(A))	30.3 (29.0 to 37.6)

^a^A higher score indicates a higher SES.

NDVI, Normalized Difference Vegetation Index; OP^DTT^, dithiothreitol; OP^ESR^, electron spin resonance; SES, socioeconomic status.

The Spearman correlations between the green space indicators, air pollutants, and traffic noise are shown in Table S3; http://links.lww.com/EE/A123). Road traffic noise levels were moderately positively correlated with the various air pollutants, with the highest correlations for PM_2.5_ absorbance (r = 0.46) and PM_10_ (r = 0.47). Correlations between the green space indicators and traffic noise ranged from −0.36 to −0.17 and the correlations between the green space indicators and air pollutants ranged from −0.72 to −0.17.

The effect estimates from the single-exposure models were similar across the four regression models, that is, the adjustment for potential confounders hardly changed the associations (Table 2). A higher average NDVI in a buffer of 3,000 m was associated with a larger diurnal decrease in cortisol concentrations {difference −0.11 nmol/L/hr (95% confidence interval [CI] = −0.21, 0.00 nmol/L/hr) per IQR increase in model 4}. Similarly, we found relationships between the total percentage of green space in a buffer of 3,000 m and a larger diurnal decrease in cortisol levels (difference −0.13 nmol/L/hr [95% CI = −0.26, 0.00 nmol/L/hr] per IQR increase in model 4). The associations with the total percentage of green space in a buffer of 3,000 m were driven by associations with the percentage of agricultural green space (difference −0.16 nmol/L/hr [95% CI = −0.35, 0.04 nmol/L/hr] per IQR increase in model 4). The average NDVI in a buffer of 300 m was also related to a larger diurnal decrease in cortisol concentrations. This relationship, however, was weaker than the relationships with green space in a buffer of 3,000 m and did not reach statistical significance. We did not find associations of ambient air pollution or traffic noise with the diurnal cortisol slope (Table 2).

**Table 2.** Associations of green space, air pollution, and traffic noise with the diurnal cortisol slope (in nmol/L per hour) at age 12 years.

Exposure (increment)	Model 1^a^	Model 2^b^	Model 3^c^	Model 4^d^
	β (95% CI)	β (95% CI)	β (95% CI)	β (95% CI)
Average NDVI in 300 m (0.13)	−0.07 (−0.16, 0.03)	−0.09 (−0.18, 0.01)	−0.08 (−0.18, 0.01)	−0.07 (−0.18, 0.03)
Total percentage of green space in 300 m (27.23)	−0.01 (−0.08, 0.07)	−0.02 (−0.10, 0.06)	−0.02 (−0.10, 0.06)	−0.01 (−0.09, 0.08)
Urban green in 300 m (7.40)	−0.02 (−0.10, 0.05)	−0.03 (−0.10, 0.05)	−0.03 (−0.10, 0.05)	−0.03 (−0.10, 0.05)
Agricultural green in 300 m (21.41)	−0.01 (−0.08, 0.05)	−0.03 (−0.09, 0.04)	−0.02 (−0.09, 0.04)	−0.01 (−0.08, 0.06)
Natural green in 300 m (yes vs. no)	0.08 (−0.10, 0.27)	0.06 (−0.13, 0.24)	0.06 (−0.13, 0.25)	0.06 (−0.12, 0.25)
Average NDVI in 3,000 m (0.13)	**−0.10 (−0.20, −0.01**)	**−0.11 (−0.21, −0.02**)	**−0.12 (−0.21, −0.02**)	**−0.11 (−0.21, 0.00**)
Total percentage of green space in 3,000 m (31.29)	**−0.11 (−0.21, 0.00**)	**−0.12 (−0.22, −0.01**)	**−0.12 (−0.22, −0.02**)	−0.13 (−0.26, 0.00)
Urban green in 3,000 m (3.84)	−0.03 (−0.16, 0.10)	−0.01 (−0.14, 0.12)	0.00 (−0.14, 0.13)	−0.01 (−0.14, 0.12)
Agricultural green in 3,000 m (36.74)	−0.14 (−0.31, 0.03)	−0.14 (−0.31, 0.04)	−0.14 (−0.31, 0.04)	−0.16 (−0.35, 0.04)
Natural green in 3,000 m (8.88)	−0.02 (−0.09, 0.04)	−0.02 (−0.09, 0.04)	−0.02 (−0.09, 0.04)	−0.03 (−0.09, 0.04)
NO_2_ (8.49 µg/m^3^)	0.03 (−0.06, 0.12)	0.05 (−0.04, 0.14)	0.05 (−0.05, 0.15)	0.02 (−0.10, 0.14)
PM_2.5_ absorbance (0.27 × 10^−5^/m)	0.00 (−0.08, 0.09)	0.03 (−0.05, 0.12)	0.03 (−0.05, 0.12)	0.00 (−0.10, 0.10)
PM_10_ (0.96 µg/m^3^)	0.01 (−0.06, 0.08)	0.03 (−0.04, 0.10)	0.03 (−0.04, 0.10)	0.01 (−0.07, 0.09)
PM_2.5_ (1.13 µg/m^3^)	−0.06 (−0.18, 0.06)	−0.02 (−0.14, 0.10)	−0.03 (−0.15, 0.09)	−0.06 (−0.19, 0.07)
OP^ESR^ (246.21 A.U./m^3^)	−0.04 (−0.15, 0.07)	−0.03 (−0.14, 0.09)	−0.03 (−0.15, 0.08)	−0.06 (−0.19, 0.06)
OP^DTT^ (0.26 nmol DTT/min/m^3^)	0.00 (−0.10, 0.09)	0.00 (−0.09, 0.10)	0.00 (−0.09, 0.10)	−0.02 (−0.13, 0.08)
Road traffic noise (6.90 dB(A))	−0.08 (−0.16, 0.00)	−0.06 (−0.14, 0.03)	−0.05 (−0.14, 0.03)	−0.08 (−0.16, 0.01)
Railway noise (8.60 dB(A))	0.01 (−0.07, 0.09)	0.01 (−0.07, 0.09)	0.01 (−0.08, 0.09)	0.00 (−0.08, 0.08)

Associations are shown for an IQR increase in exposure, except for natural green in a buffer of 300 m.

Associations with the percentages of urban, agricultural, and natural green space are adjusted for the other types of green space in the same buffer size (plus additional confounders as detailed in footnotes a–d).

Statistically significant results are highlighted in bold (*P* < 0.05).

^a^Unadjusted model.

^b^Adjusted for sex, age, parental level of education, maternal smoking during pregnancy, smoking in the child’s home, pubertal development, height, and season.

^c^Includes model 2 and neighborhood SES.

^d^Includes model 2 and degree of urbanization (in two categories: urban area and nonurban area).

CI indicates confidence interval; NDVI, Normalized Difference Vegetation Index; OP^DTT,^ dithiothreitol; OP^ESR^, electron spin resonance; SES, socioeconomic status.

We found no evidence for effect modification by parental level of education (*P* values between 0.09 and 0.89; Table S4; http://links.lww.com/EE/A123). We also observed no significant interactions between the degree of urbanization and the average NDVI and total percentage of green space (*P* values between 0.15 and 0.87). However, associations of green space in a buffer of 3,000 m with the diurnal cortisol slope were stronger in children living in nonurban areas than in children living in urban areas (Table S5; http://links.lww.com/EE/A123).

Relationships between the total percentage of green space in a buffer of 3,000 m and the diurnal cortisol slope were slightly stronger in three-exposure models (for example, difference −0.19 nmol/L/hr [95% CI = −0.34, −0.04 nmol/L/hr] per IQR increase after additional adjustment for OP^DTT^ and road traffic noise in model 4; Table 3). Associations with the average NDVI in a buffer of 3,000 m hardly changed after adjustment for air pollution and road traffic noise.

**Table 3.** Associations of green space in a 3,000 m buffer with the diurnal cortisol slope (in nmol/L per hour) at age 12 years, adjusted for air pollution and road traffic noise.

Adjusted for^a^	Average NDVI in 3,000 m	Total percentage of green space in 3,000 m
	β (95% CI)	β (95% CI)
NO_2_ + road traffic noise	**−0.12 (−0.24, 0.00**)	−0.15 (−0.32, 0.01)
PM_2.5_ absorbance + road traffic noise	**−0.11 (−0.23, 0.00**)	−0.15 (−0.30, 0.00)
PM_10_ + road traffic noise	**−0.11 (−0.22, 0.00**)	−0.14 (−0.29, 0.01)
PM_2.5_ + road traffic noise	**−0.12 (−0.22, −0.01**)	**−0.15 (−0.29, −0.01**)
OP^ESR^ + road traffic noise	**−0.12 (−0.22, −0.01**)	**−0.15 (−0.29, −0.02**)
OP^DTT^ + road traffic noise	**−0.15 (−0.27, −0.04**)	**−0.19 (−0.34, −0.04**)

Associations are shown for an IQR increase in exposure.

Statistically significant results are highlighted in bold (*P* < 0.05).

^a^Additionally adjusted for sex, age, parental level of education, maternal smoking during pregnancy, smoking in the child’s home, pubertal development, height, season, and degree of urbanization (in two categories: urban area and nonurban area).

CI indicates confidence interval; NDVI, Normalized Difference Vegetation Index; OP^DTT^, dithiothreitol; OP^ESR^, electron spin resonance.

We did not observe associations of green space in buffers of 500 m and 1000 m with the diurnal cortisol slope (e.g., difference −0.03 nmol/L/hr [95% CI −0.14, 0.08 nmol/L/hr] per IQR increase in the average NDVI in a 1000 m buffer in the full study population; Table S6; http://links.lww.com/EE/A123). Excluding children who had moved in the 2 years preceding the collection of the saliva samples did not influence the results (Table S7; http://links.lww.com/EE/A123). Finally, after the exclusion of children whose saliva samples had not been stored according to protocol, the associations of green space in a buffer of 3,000 m with the diurnal cortisol slope remained (Table S8; http://links.lww.com/EE/A123).

## Discussion

### Main findings

We found that higher exposure to residential green space in a buffer of 3,000 m was associated with a larger diurnal decrease in saliva cortisol concentrations in children 12 years of age. These associations were largely driven by associations with the percentage of agricultural green space and by associations in children living in nonurban areas. The relationships between green space and the diurnal cortisol slope remained after adjustment for ambient air pollution and road traffic noise. We observed no associations of ambient air pollution or traffic noise with the diurnal cortisol slope.

### Comparison with previous epidemiologic studies and interpretation of our findings

Several epidemiologic studies have shown that higher exposure to green space is related to lower self-reported stress levels in children and adolescents.^6,7,17^ Only one previous study has examined associations of green space with cortisol concentrations in children. This study by Van Aart et al^17^ found no relation between residential greenness (defined as the percentage of semi-natural and forested area in a buffer of 2,000 m and the percentage of agricultural area in a buffer of 300 m) and hair cortisol concentrations, an indicator of stress exposure during the last 3 months, in children 9–15 years of age in Belgium.

We found a more consistent association of a larger diurnal decrease in cortisol concentrations with green space in a buffer of 3,000 m than with green space in buffers of 300 m, 500 m, and 1,000 m. This may imply that the availability of green space in a greater area surrounding the children’s homes, rather than green space closer to home, is related to lower stress levels in our study population. In the Netherlands, children 12 years of age generally have a high level of independence (i.e., they are allowed to visit places further away from home) and cycle to school independently. This may explain why green space in a buffer of 3,000 m was more closely related to the diurnal cortisol slope than green space in smaller buffers in this study.

Green space may reduce stress directly, that is, by providing opportunities to escape from physical and social stressors, or by promoting physical activity, increasing social cohesion, and reduced exposure to environmental stressors.^1–3^ In our study, the associations of green space in a buffer of 3,000 m with a larger diurnal cortisol slope remained after adjustment for ambient air pollution and road traffic noise. This indicates that the relationships between green space and the diurnal cortisol slope are not explained by lower air pollution or road traffic noise levels (as a result of fewer air pollution and noise sources in green areas). Van Aart et al^17^ found associations of residential greenness with lower self-reported psychosocial stress independent of ambient concentrations of black carbon and PM_2.5_ and traffic noise levels, which is consistent with the findings from our study. Future studies are needed that examine the contribution of the different potential pathways between green space and stress levels in children. These studies should assess associations of specific types of green space, including agricultural green space, and include information on the use of green spaces by the study participants.

We did not observe associations between ambient air pollution and the diurnal cortisol slope in children 12 years of age. A previous study found that ambient NO_2_ exposure, but not PM_2.5_ exposure, was associated with a decrease in the diurnal cortisol slope in 140 adolescents in Los Angeles.^19^ NO_2_ concentrations and the variability in NO_2_ concentrations were higher in the study in Los Angeles than in our study (mean [IQR] 44.2 [10.0] µg/m^3^ vs. 22.5 [8.5] µg/m^3^ in our study), which may explain the discrepancy between the two studies.

In this study, no associations between traffic noise exposure and the diurnal cortisol slope were observed. No previous studies have examined relationships between road traffic or railway noise exposure and the diurnal cortisol slope in children or adolescents. However, three small cross-sectional studies (sample sizes from 43 to 115) showed significantly higher urinary or saliva cortisol levels in children with high road traffic noise exposure.^9^ Residential road traffic noise exposure was not associated with morning or evening saliva cortisol concentrations in adolescents 16 years of age in a recent study in Stockholm County.^18^ In the same study, noise annoyance (mainly due to noise from neighbors or road traffic at the residence) was related to higher morning cortisol levels.^18^ This suggests that individual perception of noise from different sources, rather than estimated road traffic noise levels, may influence cortisol concentrations in children. In our study, no information on noise annoyance was available.

### Strengths and limitations

This study has several strengths. We have measured a commonly used biomarker of chronic stress in a large population of children 12 years of age. We estimated exposure to multiple spatially correlated environmental factors that may be associated with stress levels in children. Furthermore, we included detailed and specific indicators of residential exposure to green space. Most previous epidemiologic studies only used the total percentage of green space or average NDVI in several buffers around participants’ home addresses to assess exposure to green space.^2^ We additionally examined associations of specific types of green space (urban, agricultural, and natural) with the diurnal cortisol slope in children.

This study also has limitations. As in most previous environmental health studies, saliva samples were only collected on 1 day. Since we only had access to 1 day of saliva cortisol data, we assumed that the collection day represented a typical circadian cycle for the participants and that the observed associations reflected long-term effects rather than acute HPA-axis modifications. One study showed that, of the cortisol features, total daily cortisol output may be most stable over time in children and adolescents, followed by the diurnal cortisol slope and the CAR.^35^ However, stability estimates were generally quite modest.^35^ Additionally, Rotenberg et al^36^ found that the diurnal cortisol profile was relatively stable in children and adolescents in Montreal, but also reported that at least 3–7 days of saliva collections are needed to minimize within-subject variance in the diurnal cortisol slope.

Children in the PIAMA study were recruited from the general population, but children of higher educated parents were somewhat overrepresented. 25.9% of the baseline PIAMA study population was available for the present study (Figure S2; http://links.lww.com/EE/A123). There was selective nonparticipation of children with lower paternal and maternal levels of education (Table S2; http://links.lww.com/EE/A123). Associations with the diurnal cortisol slope hardly changed after adjustment for parental level of education and we found no evidence for effect modification by parental level of education. We therefore assume that the associations of green space, ambient air pollution, and traffic noise with the diurnal cortisol slope would not be different in the general population of Dutch children.

Furthermore, we did not include exposure to green space, ambient air pollution, and traffic noise at the school addresses, where children also spend a considerable amount of time. A study by Bilenko et al^37^ has shown that ambient air pollution concentrations (r = 0.71–0.91), but not traffic noise levels (r = 0.34), at home and school addresses were highly correlated at age 12 years in the PIAMA study. This implies that the inclusion of residential exposures only may have led to misclassification of individual traffic noise exposure, but that measurement error resulting from reliance on residential exposure to air pollution is likely small.

We used the average NDVI of 2010, LUR models that were based on measurement campaigns in 2009 and traffic noise estimates for 2011 to assess residential exposure to green space, air pollution, and traffic noise at the time of the medical examination at age 12 years (from 2008 to 2010). The temporal misalignment between the exposures and outcome may have caused bias in our observed exposure-response relationships. However, we found high correlations between the average NDVI in 2000/2002 and 2010 for the home addresses of participants of the PIAMA study (r = 0.87 for the average NDVI in 300 m; r = 0.91 for the average NDVI in 3,000 m). This indicates that the spatial contrasts in green space levels in the Netherlands remain stable over a period of 10 years. Additionally, multiple studies from Europe have shown that the spatial variation in ambient air pollution or noise levels remained stable over periods of 7–12 years.^21,38–40^

Like the majority of previous epidemiologic studies, we only had information on traffic noise levels outside the homes of our study participants. We lacked information on window type, orientation of the bedroom, and indoor insulation, which may affect a child’s actual exposure to traffic noise. Another limitation is that we did not know if and how often our study participants used the green spaces in the specified buffers around their homes. Finally, information on the quality of green spaces was not available in this study. Quality characteristics of green spaces, such as safety, walkability, and sport/play facilities, may affect the use of green spaces.^3,41^

### Implications and future research directions

High levels of stress during childhood have been linked to impaired emotional and behavioral development as well as adverse health consequences later in life, including depression, cardiovascular disease, and diabetes.^42^ It is therefore important to assess the impact of modifiable determinants on chronic stress levels in children. The results of this study suggest that protecting or increasing green spaces may be effective public health interventions to reduce stress levels in children in the Netherlands. However, more epidemiologic studies are needed that assess associations of green space with both subjective stress and cortisol concentrations in children to design and implement effective public health interventions. Future studies should examine associations of green space, air pollution, and traffic noise with saliva cortisol concentrations, and other (physiologic) markers of chronic stress, that are collected during multiple days in children and adolescents, taking other sources of acute and chronic stress into account as potential confounders.

## Conclusions

Residential exposure to green space in a buffer of 3,000 m may be associated with a larger diurnal decrease in saliva cortisol concentrations, an indicator of lower chronic stress levels, in children 12 years of age. Ambient air pollution and traffic noise were not related to the diurnal cortisol slope in children.

## Conflicts of interest statement

The authors declare that they have no conflicts of interest with regard to the content of this report.

## ACKNOWLEDGMENTS

We like to thank Piet Beekhof for analyzing the saliva samples.

## Supplementary Material


