# Supplementary material for: Green space, air pollution, traffic noise and saliva cortisol in children: The PIAMA study
Source: Environ Epidemiol. 2021 Apr 2;5(2):e141. doi: 10.1097/EE9.0000000000000141 (PMC8043724; doi:10.1097/EE9.0000000000000141)
Supplement: Supplementary file 1 [file ee9-5-e141-s001.docx]

Supplemental Material

**Green space, air pollution, traffic noise and saliva cortisol in children: The PIAMA study**

Lizan D. Bloemsma, Alet H. Wijga, Jochem O. Klompmaker, Gerard Hoek, Nicole A.H. Janssen, Marieke Oldenwening, Gerard H. Koppelman, Erik Lebret, Bert Brunekreef, Ulrike Gehring

**Table of contents**

| **Figure S1.** Locations of the antenatal clinics that recruited pregnant women in 1996/1997. | 2 |
| --- | --- |
| **Figure S2.** Flow diagram of study participants. | 3 |
| **Figure S3.** Exposure-response curves for the associations of green space, air pollution and traffic noise with the diurnal cortisol slope at age 12 years. | 4 |
| **Figure S4.** Distributions of saliva cortisol concentrations (in nmol/L) 30 minutes after awakening and at 8.00pm in 1027 children aged 12 years. | 6 |
| **Table S1.** Spearman correlations between the average NDVI in buffers of 100m, 300m, 500m, 1000m and 3000m. | 7 |
| **Table S2.** Comparison between children included in this study and the baseline PIAMA study population. | 8 |
| **Table S3.** Spearman correlations between green space, ambient air pollution and traffic noise at age 12 years. | 9 |
| **Table S4.** Associations of green space, air pollution and traffic noise with the diurnal cortisol slope, stratified by parental level of education (low/intermediate vs. high). | 10 |
| **Table S5.** Associations of the average NDVI and total percentage of green space with the diurnal cortisol slope (in nmol/L per hour), stratified by degree of urbanization. | 11 |
| **Table S6.** Associations of green space in buffers of 500m and 1000m with the diurnal cortisol slope (in nmol/L/hr) at age 12 years. | 12 |
| **Table S7.** Associations of green space, air pollution and traffic noise with the diurnal cortisol slope (in nmol/L/hr) in children who had not moved in the two years preceding the collection of the saliva samples (n=938). | 13 |
| **Table S8.** Associations of green space, air pollution and traffic noise with the diurnal cortisol slope (in nmol/L/hr) in children whose samples have been stored according to protocol (n=921). | 14 |


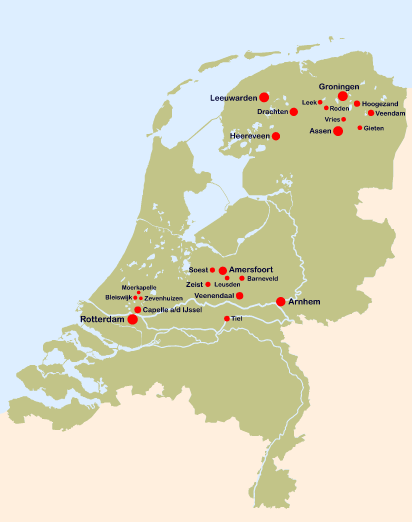


Figure S1. Locations of the antenatal clinics that recruited pregnant women in 1996/1997.

**1996/1997: Baseline PIAMA study population**
3963 children

80%

**2008-2010: Medical examination at age 12 years**

3169 children invited

48%

1511 children participated in medical examination

72%

1094 children collected saliva samples during medical examination

Exclusion: children for whom ≥1 sample did not contain enough saliva to determine cortisol concentrations (n=24), and children whose samples were unintentionally mixed or did not have a cap (n=3)

2%

Exclusion: extremely high cortisol concentrations (n=5), children with diabetes (n=3), and children who used corticosteroids on the day of the saliva collection (n=32)

4%

1027 children included in present study

Figure S2. Flow diagram of study participants.


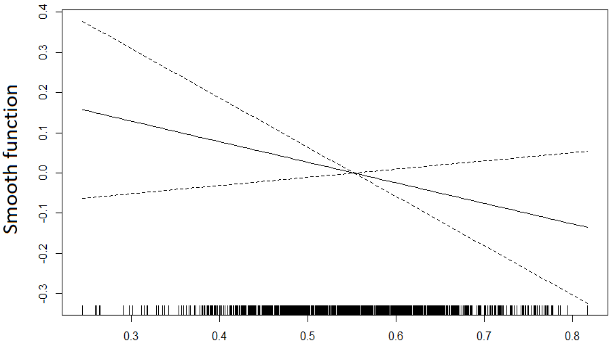

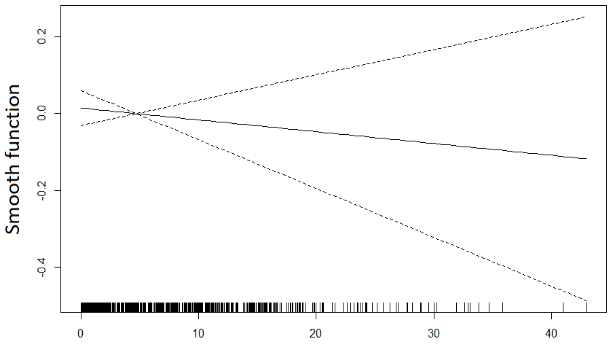

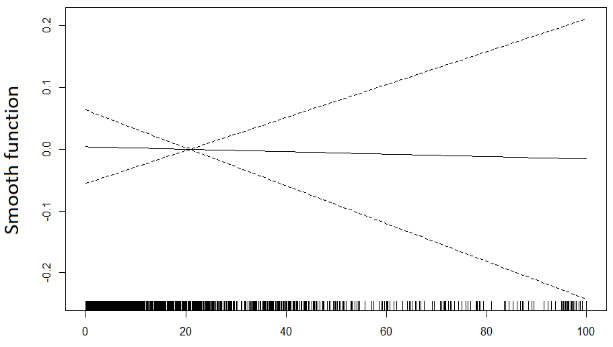


NDVI in 300m Total green space in 300m Urban green in 300m


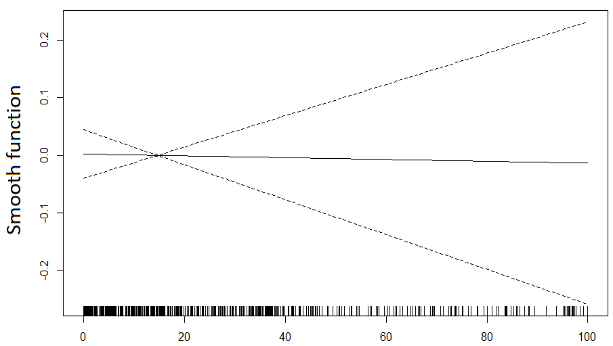


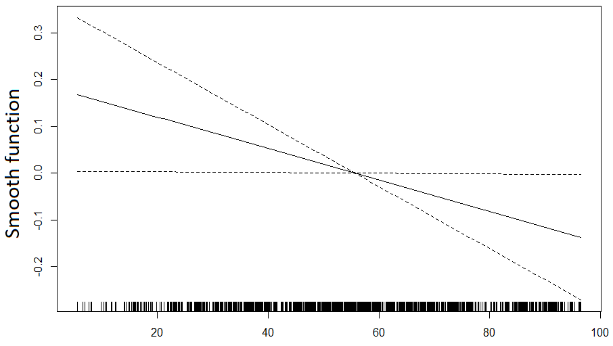

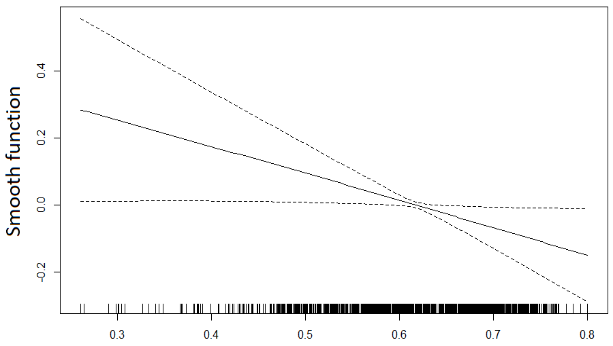


Agricultural green in 300m NDVI in 3000m Total green space in 3000m


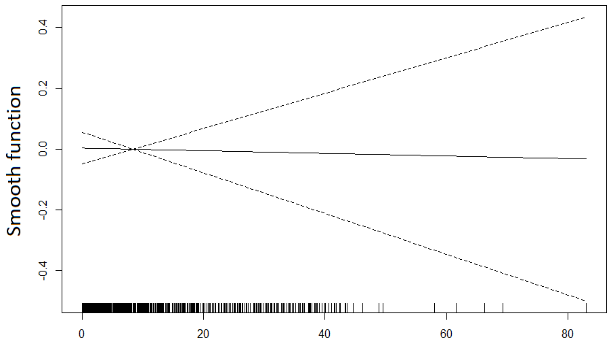

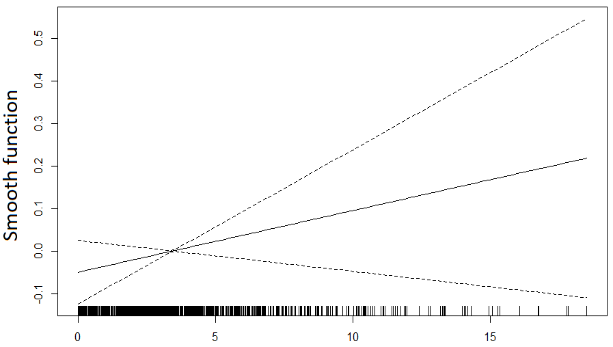

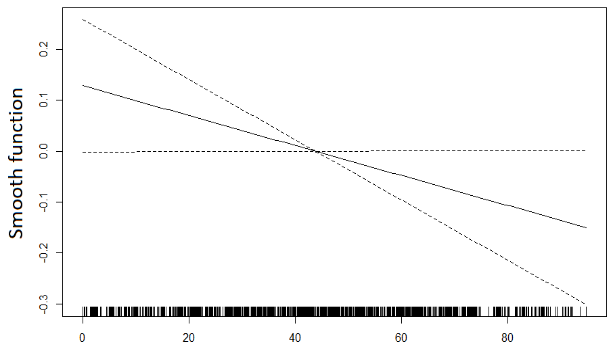


Urban green in 3000m Agricultural green in 3000m Natural green in 3000m

Figure S3. Exposure-response curves for the associations of green space, air pollution and traffic noise with the diurnal cortisol slope at age 12 years.
The x-axes show the number of observations for each value of the environmental exposures.


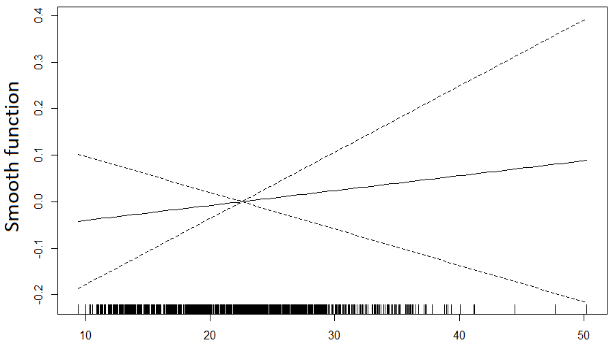


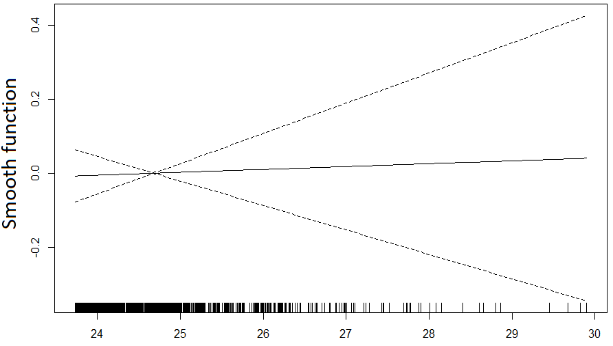

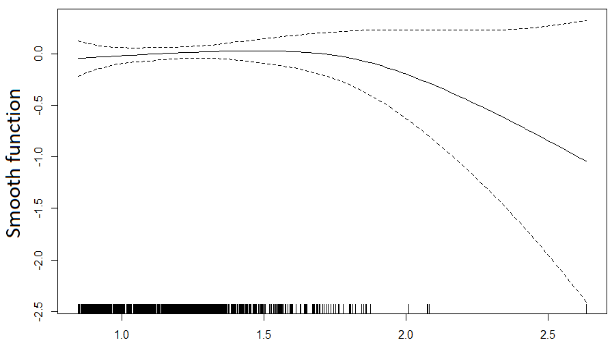


NO_2_ PM_2.5_ absorbance PM_10_


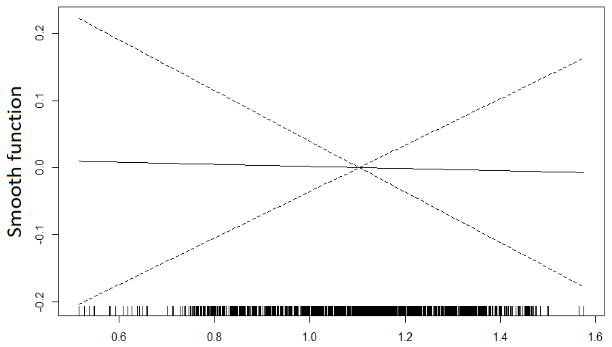

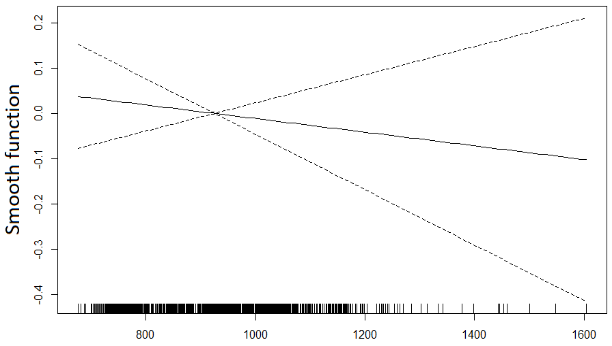

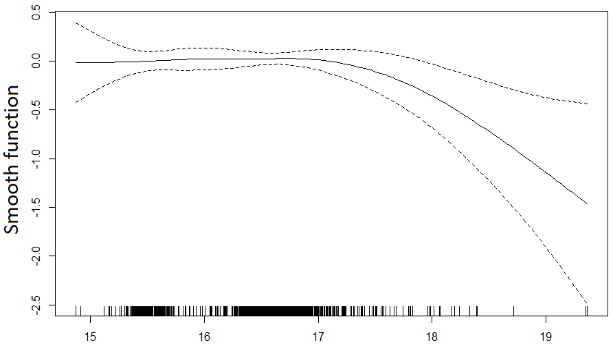


PM_2.5_ OP^ESR^ OP^DTT^


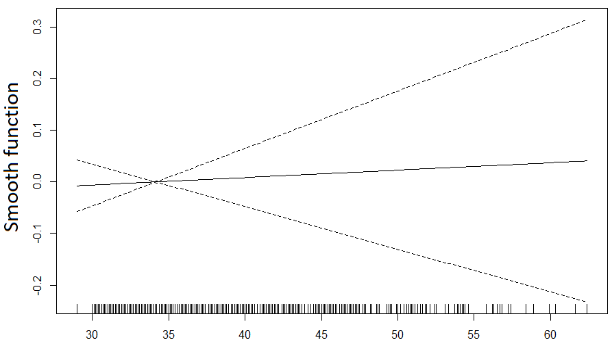

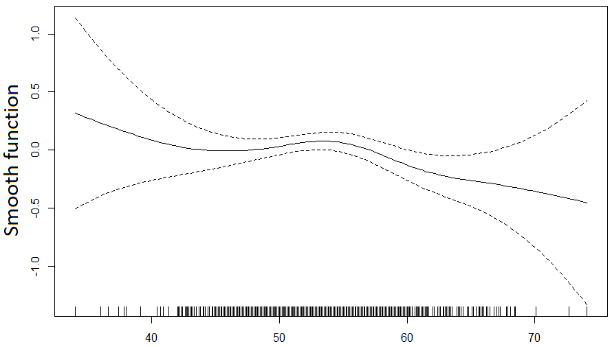


Road traffic noise Railway noise

Figure S3 (continued).


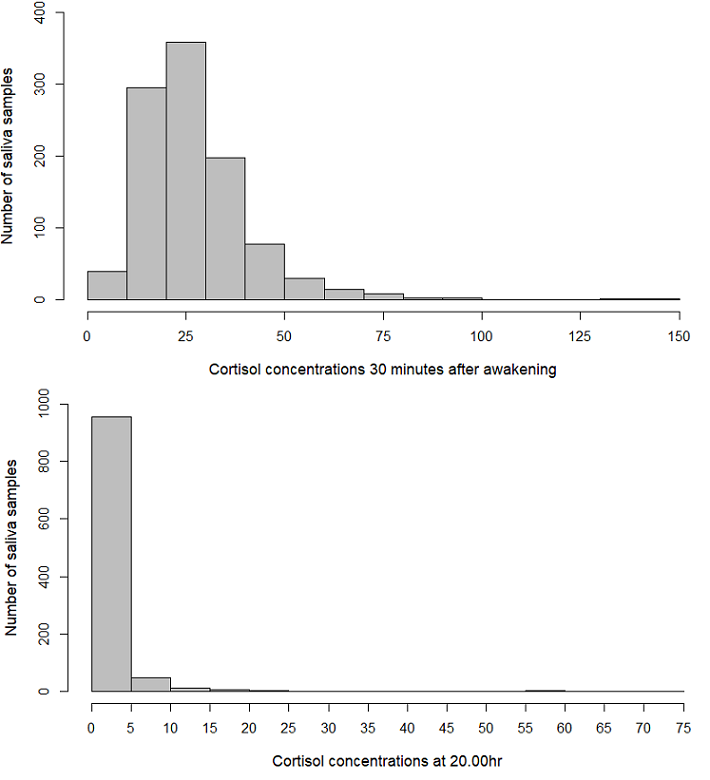


Figure S4. Distributions of saliva cortisol concentrations (in nmol/L) 30 minutes after awakening and at 8.00pm in 1027 children aged 12 years.

Table S1. Spearman correlations between the average NDVI in buffers of 100m, 300m, 500m, 1000m and 3000m.

|  | **100m** | **300m** | **500m** | **1000m** | **3000m** |
| --- | --- | --- | --- | --- | --- |
| **100m** |  | 0.83 | 0.74 | 0.63 | 0.50 |
| **300m** |  |  | 0.93 | 0.78 | 0.59 |
| **500m** |  |  |  | 0.88 | 0.65 |
| **1000m** |  |  |  |  | 0.77 |
| **3000m** |  |  |  |  |  |

Abbreviations: NDVI = Normalized Difference Vegetation Index.

All correlation coefficients are statistically significant (p<0.05).

Table S2. Comparison between children included in this study and the baseline PIAMA study population.

| **Characteristic** | **Children included in this study (n=1027)** | **Baseline PIAMA study population (n=3963)** |
| --- | --- | --- |
| Sex, n (%)  Boy  Girl | 504 (49.1)  523 (50.9) | 2054 (51.8)  1908 (48.2) |
| Maternal level of education, n (%)  Low  Intermediate  High | 186 (18.2)  413 (40.3)  426 (41.6) | 894 (23.5)  1582 (41.6)  1331 (35.0) |
| Paternal level of education, n (%)  Low  Intermediate  High | 206 (20.2)  337 (33.1)  475 (46.7) | 973 (25.9)  1295 (34.4)  1493 (39.7) |
| Maternal smoking during pregnancy, n (%)  Yes  No | 135 (13.3)  884 (86.8) | 703 (17.9)  3217 (82.1) |
| Region, n (%) ^a^  North  Central  West | 331 (32.2)  450 (43.8)  246 (24.0) | 1231 (31.1)  1586 (40.0)  1146 (28.9) |

^a^ North: provinces Groningen, Friesland, Drenthe; Central: provinces Utrecht, Gelderland, Flevoland; West: city of Rotterdam and surrounding municipalities.

|  | | **NDVI** | | **Total percentage of green space** | | **Air pollutants** | | | | | | **Noise** | |
| --- | --- | --- | --- | --- | --- | --- | --- | --- | --- | --- | --- | --- | --- |
|  |  | **300m** | **3000m** | **300m** | **3000m** | **NO_2_** | **PM_2.5_ abs** | **PM_10_** | **PM_2.5_** | **OP^ESR^** | **OP^DTT^** | **Road traffic** | **Railway** |
| **NDVI** | **300m** |  | 0.59 | 0.64 | 0.53 | -0.60 | -0.47 | -0.41 | -0.32 | -0.27 | -0.67 | -0.26 | -0.20 |
|  | **3000m** |  |  | 0.30 | 0.83 | -0.64 | -0.50 | -0.52 | -0.22 | -0.17 | -0.56 | -0.21 | -0.28 |
| **Total percentage green space** | **300m** |  |  |  | 0.46 | -0.46 | -0.38 | -0.38 | -0.28 | -0.33 | -0.53 | -0.21 | -0.17 |
|  | **3000m** |  |  |  |  | -0.72 | -0.64 | -0.70 | -0.34 | -0.38 | -0.57 | -0.26 | -0.36 |
| **Air pollutants** | **NO_2_** |  |  |  |  |  | 0.89 | 0.76 | 0.65 | 0.63 | 0.73 | 0.39 | 0.34 |
|  | **PM_2.5_ abs** |  |  |  |  |  |  | 0.87 | 0.81 | 0.72 | 0.55 | 0.46 | 0.34 |
|  | **PM_10_** |  |  |  |  |  |  |  | 0.63 | 0.53 | 0.49 | 0.47 | 0.31 |
|  | **PM_2.5_** |  |  |  |  |  |  |  |  | 0.80 | 0.37 | 0.41 | 0.29 |
|  | **OP^ESR^** |  |  |  |  |  |  |  |  |  | 0.30 | 0.36 | 0.30 |
|  | **OP^DTT^** |  |  |  |  |  |  |  |  |  |  | 0.25 | 0.19 |
| **Noise** | **Road traffic** |  |  |  |  |  |  |  |  |  |  |  | 0.16 |
|  | **Railway** |  |  |  |  |  |  |  |  |  |  |  |  |

Table S3. Spearman correlations between green space, ambient air pollution and traffic noise at age 12 years.

Abbreviations: NDVI = Normalized Difference Vegetation Index; OP^ESR^ = electron spin resonance; OP^DTT^ = dithiothreitol.

All correlation coefficients are statistically significant (p<0.05).

| **Exposure (increment)** | **Low/intermediate** | **High** |
| --- | --- | --- |
|  | **β (95% CI)** | **β (95% CI)** |
| Average NDVI in 300m (0.13) | -0.07 (-0.23, 0.08) | -0.07 (-0.21, 0.07) |
| Total percentage of green space in 300m (27.23) | 0.01 (-0.11, 0.13) | -0.02 (-0.14, 0.10) |
| Urban green in 300m (7.40) | 0.06 (-0.06, 0.17) | -0.08 (-0.17, 0.02) |
| Agricultural green in 300m (21.41) | 0.01 (-0.09, 0.11) | -0.02 (-0.12, 0.07) |
| Natural green in 300m (yes vs. no) | 0.09 (-0.20, 0.37) | 0.06 (-0.19, 0.30) |
| Average NDVI in 3000m (0.13) | -0.06 (-0.21, 0.08) | **-0.15 (-0.31, 0.00)** |
| Total percentage of green space in 3000m (31.29) | -0.14 (-0.33, 0.06) | -0.13 (-0.32, 0.06) |
| Urban green in 3000m (3.84) | -0.04 (-0.25, 0.18) | 0.03 (-0.15, 0.20) |
| Agricultural green in 3000m (36.74) | -0.14 (-0.43, 0.15) | -0.15 (-0.42, 0.13) |
| Natural green in 3000m (8.88) | -0.07 (-0.17, 0.02) | 0.01 (-0.08, 0.10) |
| NO_2_ (8.49 µg/m^3^) | 0.01 (-0.17, 0.18) | 0.03 (-0.13, 0.19) |
| PM_2.5_ absorbance (0.27 x 10^-5^/m) | -0.02 (-0.17, 0.13) | 0.01 (-0.13, 0.15) |
| PM_10_ (0.96 µg/m^3^) | 0.00 (-0.13, 0.13) | 0.01 (-0.10, 0.12) |
| PM_2.5_ (1.13 µg/m^3^) | -0.05 (-0.24, 0.15) | -0.08 (-0.25, 0.10) |
| OP^ESR^ (246.21 A.U./m^3^) | 0.02 (-0.15, 0.20) | -0.14 (-0.31, 0.03) |
| OP^DTT^ (0.26 nmol DTT/min/m^3^) | 0.02 (-0.14, 0.18) | -0.05 (-0.19, 0.08) |
| Road traffic noise (6.90 dB(A)) | -0.03 (-0.16, 0.11) | -0.11 (-0.23, 0.00) |
| Railway noise (8.60 dB(A)) | 0.08 (-0.05, 0.20) | -0.07 (-0.18, 0.05) |

Table S4. Associations of green space, air pollution and traffic noise with the diurnal cortisol slope, stratified by parental level of education (low/intermediate vs. high). ^a^

Abbreviations: CI = confidence interval; NDVI = Normalized Difference Vegetation Index; OP^ESR^ = electron spin resonance; OP^DTT^ = dithiothreitol.

Associations are shown for an interquartile range increase in exposure, except for natural green in a buffer of 300m.

Associations with the percentages of urban, agricultural and natural green space are adjusted for the other types of green space in the same buffer size (plus additional confounders as detailed in footnote a).
Statistically significant results are highlighted in bold (p <0.05).

^a^ Adjusted for sex, age, maternal smoking during pregnancy, smoking in the child’s home, pubertal development, height, season and degree of urbanization (in two categories: urban area; non-urban area).

Table S5. Associations of the average NDVI and total percentage of green space with the diurnal cortisol slope (in nmol/L per hour), stratified by degree of urbanization. ^a^

| **Exposure (increment)** | **Urban area (≥1500 addresses/km^2^)** | **Non-urban area (<1500 addresses/km^2^)** |
| --- | --- | --- |
|  | **β (95% CI)** | **β (95% CI)** |
| Average NDVI in 300m (0.13) | -0.08 (-0.26, 0.11) | -0.06 (-0.19, 0.07) |
| Total percentage of green space in 300m (27.23) | 0.14 (-0.10, 0.38) | -0.03 (-0.11, 0.06) |
| Average NDVI in 3000m (0.13) | -0.01 (-0.19, 0.17) | **-0.16 (-0.29, -0.03)** |
| Total percentage of green space in 3000m (31.29) | -0.05 (-0.29, 0.19) | -0.16 (-0.33, 0.01) |

Abbreviations: CI = confidence interval; NDVI = Normalized Difference Vegetation Index.

Statistically significant results are highlighted in bold (p <0.05).

^a^ Adjusted for sex, age, parental level of education, maternal smoking during pregnancy, smoking in the child’s home, pubertal development, height, season and neighborhood SES.

|  | **Full study population ^a^** | **Urban area (≥1500 addresses/km^2^) ^b^** | **Non-urban area (<1500 addresses/km^2^) ^b^** |
| --- | --- | --- | --- |
| **Exposure (increment)** | **β (95% CI)** | **β (95% CI)** | **β (95% CI)** |
| Average NDVI in 500m (0.13) | -0.03 (-0.13, 0.07) | -0.04 (-0.22, 0.14) | -0.01 (-0.14, 0.12) |
| Total percentage of green space in 500m (30.70) | 0.01 (-0.09, 0.11) | 0.11 (-0.16, 0.38) | 0.00 (-0.11, 0.10) |
| Urban green in 500m (7.83) | -0.01 (-0.10, 0.08) | N/A | N/A |
| Agricultural green in 500m (31.46) | 0.00 (-0.10, 0.11) | N/A | N/A |
| Natural green in 500m (yes vs. no) | 0.04 (-0.11, 0.18) | N/A | N/A |
| Average NDVI in 1000m (0.13) | -0.03 (-0.14, 0.08) | 0.02 (-0.17, 0.21) | -0.06 (-0.19, 0.08) |
| Total percentage of green space in 1000m (37.06) | 0.01 (-0.11, 0.14) | 0.14 (-0.17, 0.45) | -0.02 (-0.16, 0.13) |
| Urban green in 1000m (5.97) | 0.01 (-0.08, 0.10) | N/A | N/A |
| Agricultural green in 1000m (39.52) | 0.01 (-0.14, 0.15) | N/A | N/A |
| Natural green in 1000m (4.73) | 0.01 (-0.03, 0.06) | N/A | N/A |

Table S6. Associations of green space in buffers of 500m and 1000m with the diurnal cortisol slope (in nmol/L/hr) at age 12 years.

Abbreviations: CI = confidence interval; NDVI = Normalized Difference Vegetation Index.

Associations are shown for an interquartile range increase in exposure, except for natural green in a buffer of 500m.

Associations with the percentages of urban, agricultural and natural green space are adjusted for the other types of green space in the same buffer size (plus additional confounders as detailed in footnote a).
Statistically significant results are highlighted in bold (p <0.05).
^a^ Adjusted for sex, age, parental level of education, maternal smoking during pregnancy, smoking in the child’s home, pubertal development, height, season and degree of urbanization (in two categories: urban area; non-urban area).

^b^ Adjusted for sex, age, parental level of education, maternal smoking during pregnancy, smoking in the child’s home, pubertal development, height, season and neighborhood SES.

Table S7. Associations of green space, air pollution and traffic noise with the diurnal cortisol slope (in nmol/L/hr) in children who had not moved in the two years preceding the collection of the saliva samples (n=938). ^a^

| **Exposure (increment)** | **β (95% CI)** |
| --- | --- |
| Average NDVI in 300m (0.13) | -0.10 (-0.21, 0.01) |
| Total percentage of green space in 300m (27.23) | -0.03 (-0.12, 0.06) |
| Urban green in 300m (7.40) | -0.03 (-0.11, 0.05) |
| Agricultural green in 300m (21.41) | -0.03 (-0.11, 0.04) |
| Natural green in 300m (yes vs. no) | 0.07 (-0.12, 0.26) |
| Average NDVI in 3000m (0.13) | **-0.11 (-0.22, 0.00)** |
| Total percentage of green space in 3000m (31.29) | **-0.16 (-0.30, -0.02)** |
| Urban green in 3000m (3.84) | -0.01 (-0.15, 0.12) |
| Agricultural green in 3000m (36.74) | -0.20 (-0.41, 0.01) |
| Natural green in 3000m (8.88) | -0.03 (-0.10, 0.04) |
| NO_2_ (8.49 µg/m^3^) | 0.03 (-0.09, 0.16) |
| PM_2.5_ absorbance (0.27 x 10^-5^/m) | 0.01 (-0.09, 0.12) |
| PM_10_ (0.96 µg/m^3^) | 0.02 (-0.07, 0.11) |
| PM_2.5_ (1.13 µg/m^3^) | -0.05 (-0.18, 0.09) |
| OP^ESR^ (246.21 A.U./m^3^) | -0.05 (-0.18, 0.08) |
| OP^DTT^ (0.26 nmol DTT/min/m^3^) | 0.00 (-0.11, 0.10) |
| Road traffic noise (6.90 dB(A)) | -0.08 (-0.18, 0.01) |
| Railway noise (8.60 dB(A)) | 0.00 (-0.09, 0.09) |

Abbreviations: CI = confidence interval; NDVI = Normalized Difference Vegetation Index; OP^ESR^ = electron spin resonance; OP^DTT^ = dithiothreitol.

Associations are shown for an interquartile range increase in exposure, except for natural green in a buffer of 300m.

Associations with the percentages of urban, agricultural and natural green space are adjusted for the other types of green space in the same buffer size (plus additional confounders as detailed in footnote a).
Statistically significant results are highlighted in bold (p <0.05).

^a^ Adjusted for sex, age, parental level of education, maternal smoking during pregnancy, smoking in the child’s home, pubertal development, height, season and degree of urbanization (in two categories: urban area; non-urban area).

Table S8. Associations of green space, air pollution and traffic noise with the diurnal cortisol slope (in nmol/L/hr) in children whose samples have been stored according to protocol (n=921). **^a^**

| **Exposure (increment)** | **β (95% CI) ^b^** |
| --- | --- |
| Average NDVI in 300m (0.13) | -0.07 (-0.18, 0.05) |
| Total percentage of green space in 300m (27.23) | 0.01 (-0.08, 0.10) |
| Urban green in 300m (7.40) | -0.02 (-0.10, 0.06) |
| Agricultural green in 300m (21.41) | 0.00 (-0.08, 0.07) |
| Natural green in 300m (yes vs. no) | 0.07 (-0.13, 0.27) |
| Average NDVI in 3000m (0.13) | **-0.12 (-0.23, -0.01)** |
| Total percentage of green space in 3000m (31.29) | **-0.15 (-0.30, -0.01)** |
| Urban green in 3000m (3.84) | 0.01 (-0.14, 0.15) |
| Agricultural green in 3000m (36.74) | -0.16 (-0.38, 0.05) |
| Natural green in 3000m (8.88) | -0.03 (-0.10, 0.04) |
| NO_2_ (8.49 µg/m^3^) | 0.05 (-0.08, 0.18) |
| PM_2.5_ absorbance (0.27 x 10^-5^/m) | 0.04 (-0.07, 0.15) |
| PM_10_ (0.96 µg/m^3^) | 0.05 (-0.04, 0.14) |
| PM_2.5_ (1.13 µg/m^3^) | -0.04 (-0.18, 0.10) |
| OP^ESR^ (246.21 A.U./m^3^) | -0.04 (-0.17, 0.09) |
| OP^DTT^ (0.26 nmol DTT/min/m^3^) | -0.02 (-0.13, 0.09) |
| Road traffic noise (6.90 dB(A)) | -0.05 (-0.14, 0.04) |
| Railway noise (8.60 dB(A)) | 0.00 (-0.08, 0.09) |

Abbreviations: CI = confidence interval; NDVI = Normalized Difference Vegetation Index; OP^ESR^ = electron spin resonance; OP^DTT^ = dithiothreitol.

Associations are shown for an interquartile range increase in exposure, except for natural green in a buffer of 300m.

Associations with the percentages of urban, agricultural and natural green space are adjusted for the other types of green space in the same buffer size (plus additional confounders as detailed in footnote b).
Statistically significant results are highlighted in bold (p <0.05).
**^a^** Children whose saliva samples were not stored in a refrigerator for a few days, children whose samples were removed from the freezer for 24 hours and children whose samples have not been stored in the freezer within seven days were excluded.

^b^ Adjusted for sex, age, parental level of education, maternal smoking during pregnancy, smoking in the child’s home, pubertal development, height, season and degree of urbanization (in two categories: urban area; non-urban area).
